# Catheter Ablation for Channelopathies: When Is Less More?

**DOI:** 10.3390/jcm13082384

**Published:** 2024-04-19

**Authors:** Adhya Mehta, Rishi Chandiramani, Binita Ghosh, Babken Asatryan, Adrija Hajra, Andreas S. Barth

**Affiliations:** 1 Department of Internal Medicine, Albert Einstein College of Medicine/Jacobi Medical Center, Bronx, NY 10461, USA; 2 Department of Medicine, Division of Cardiology, Johns Hopkins University School of Medicine, Baltimore, MD 21287, USA; 3 Department of Internal Medicine, SSM Health St. Mary Hospital, St. Louis, MO 63117, USA; binita.ghosh@ssmhealth.com; 4 Department of Internal Medicine, Brigham and Women’s Hospital, Boston, MA 02115, USA

**Keywords:** channelopathies, long QT syndrome, Brugada syndrome, ventricular fibrillation, cardiac ablation

## Abstract

Ventricular fibrillation (VF) is a common cause of sudden cardiac death in patients with channelopathies, particularly in the young population. Although pharmacological treatment, cardiac sympathectomy, and implantable cardioverter defibrillators (ICD) have been the mainstay in the management of VF in patients with channelopathies, they are associated with significant adverse effects and complications, leading to poor quality of life. Given these drawbacks, catheter ablation has been proposed as a therapeutic option for patients with channelopathies. Advances in imaging techniques and modern mapping technologies have enabled increased precision in identifying arrhythmia triggers and substrate modification. This has aided our understanding of the underlying pathophysiology of ventricular arrhythmias in channelopathies, highlighting the roles of the Purkinje network and the epicardial right ventricular outflow tract in arrhythmogenesis. This review explores the role of catheter ablation in managing the most common channelopathies (Brugada syndrome, congenital long QT syndrome, short QT syndrome, and catecholaminergic polymorphic ventricular tachycardia). While the initial results for ablation in Brugada syndrome are promising, the long-term efficacy and durability of ablation in different channelopathies require further investigation. Given the genetic and phenotypic heterogeneity of channelopathies, future studies are needed to show whether catheter ablation in patients with channelopathies is associated with a reduction in VF, and psychological distress stemming from recurrent ICD shocks, particularly relative to other available therapeutic options (e.g., quinidine in high-risk Brugada patients).

## 1. Introduction

Channelopathies, including long QT syndrome (LQTS), Brugada syndrome (BrS), short QT syndrome (SQTS), and catecholaminergic polymorphic ventricular tachycardia (CPVT) account for up to 10% of sudden cardiac deaths (SCD) in young individuals. SCD is often caused by ventricular fibrillation (VF) [1,2,3]. The management of VF in channelopathies is a complex and evolving field. Conventional therapeutic strategies, such as pharmacological interventions, cardiac sympathectomy, and implantable cardioverter defibrillators (ICDs), aim to mitigate arrhythmia and SCD risk. In patients diagnosed with channelopathies, the initial strategy remains avoidance of triggers and medical management with β-blockers and antiarrhythmic drugs while implantation of ICDs is indicated in patients with arrhythmogenic syncope and SCD survivors for secondary prevention. Cardiac sympathectomy is primarily employed in patients with recurrent appropriate ICD shocks refractory to medical therapy [4]. These strategies, however, do not fully address the underlying mechanisms responsible for VF initiation and maintenance. In recent years, there has been growing interest in exploring catheter ablation (CA) to treat VF in patients with channelopathies. While CA is effective in treating monomorphic ventricular tachycardia (VT), particularly when associated with structural heart disease or scar tissue, the success rates for VF ablation are generally lower than those for monomorphic VT. As VF is due to functional reentry that involves the entire ventricular myocardium and does not have a specific focal point or circuit that can be easily targeted, VF is more challenging to manage with ablation than monomorphic VT due to scars from myocardial infarction or replacement fibrosis. Ablation requires a thorough understanding of the exact mechanisms and triggers of VF and the underlying substrate, which vary among various channelopathies and from patient to patient. This review discusses the rationale, mechanisms, challenges, and emerging concepts associated with ablation strategies in the context of channelopathies.

## 2. Mechanistic Insights into the Substrate and Triggers of VF

The mechanism of VF in channelopathies is complex and can vary depending on the specific channelopathy involved. In LQTS, early afterdepolarizations (EADs) are the most likely arrhythmogenic triggers for the initiation of VF, which typically presents as polymorphic VT or torsades de pointes (TdP) [5,6]. EADs are associated with prolonged action potential duration (APD), resulting from the inhibition of delayed rectifier potassium currents or delayed inactivation of calcium (I_CaL_) or sodium (I_Na_) currents during the plateau phase. EADs can induce VF by 1) triggered activity whereby EADs serve as premature beats that trigger sustained arrhythmias when they coincide with the vulnerable period of the cardiac cycle or by 2) dispersion of repolarization, creating areas of the myocardium with delayed repolarization, which can serve as reentry pathways for VF. The onset of ventricular arrhythmia (VA) in LQTS also varies depending on the specific genetic subtype. LQTS1 and LQTS2 are caused by pathogenic variants in the potassium channel genes *KCNQ1* and *KCNH2,* respectively. These genes encode for the α-subunits of the slow and rapid delayed rectifier potassium channels (I_Ks_ and I_Kr_, respectively), which are the main channels responsible for the repolarization phase of the cardiac action potential. Loss of function variants in *KCNQ1* or *KCNH2* results in dysfunctional or reduced expression of these channels, leading to delayed repolarization of the cardiac myocytes [7,8]. LQTS3 is due to gain-of-function variants in the *SCN5A* gene, causing an increased inward sodium current during the plateau phase of the action potential [9]. In LQTS1-3, the mechanisms underlying arrhythmogenesis involve the imbalance between inward and outward currents, i.e., either less outward currents or more inward currents during the cardiac action potential, resulting in prolonged repolarization and increased susceptibility to triggered activity and reentry arrhythmias. Individuals with LQTS1 typically experience arrhythmias during periods of tachycardia following adrenergic stress, whereas patients with LQTS2 and LQTS3 are most vulnerable to developing TdP after sinus pauses and periods of bradycardia, respectively. It is currently unclear which region of the myocardium contributes the most to the initiation of TdP and VF, and whether there is such a region with predominant involvement in the first place. Transmural differences in cellular electrophysiology have long been recognized with longer action potentials in the subendocardium than in subepicardial cell layers. These physiological transmural differences in action potentials across the myocardial wall contribute to the establishment of transmural voltage gradients, which are essential for normal cardiac function and the prevention of arrhythmias. When this physiologic transmural voltage gradient is disrupted, transmural dispersion of repolarization arises, which plays a significant role in the pathophysiology of LQTS and BrS. A “spike and dome” morphology in transmembrane action potential has been observed in epicardial cells but not in the endocardium. The difference is in part from the transient outward current (I_to_), which is a main contributor to phase 1 of depolarization and exhibits a larger cell density in subepicardial cell layers compared to the subendocardium [10]. In BrS, the loss of function of the sodium channel *SCN5A* can lead to a large transmural voltage gradient by allowing a relatively large epicardial I_to_ to repolarize the cells selectively in subepicardial cell layers, leading to short epicardial action potentials. As the I_to_ density is much smaller in the subendocardium, the duration of the subendocardial action potentials remains relatively unchanged, giving rise to a large transmural voltage gradient, which is most pronounced in the right ventricular outflow tract (RVOT). On the electrocardiogram (ECG), this transmural voltage gradient in BrS manifests as characteristic right precordial ST-elevations.

Additionally, distinct from the surrounding ventricular muscle, the specialized conduction system, including the Purkinje fibers (PFs), exhibits different electrophysiological properties, most notably a longer action potential duration (APD) [11,12]. This adds to the complexity of the electrical landscape of the intact ventricular myocardium. Given the longer action potentials in the subendocardium and PFs, endocardial CA of triggers was initially attempted in patients with LQTS, demonstrating varying degrees of success [13]. More recently, CA targeting the epicardium in LQTS has been suggested [14]. Histological complexities within the epicardial area, including the presence of multipotent stem cells, can lead to fibrous and fatty infiltration in the subepicardium [14,15]. This microstructural remodeling may lead to the slowing of conduction by disrupting physiologic myocyte-to-myocyte coupling, creating a potential pathway for the development of reentrant arrhythmias and altering the physiologic transmural repolarization gradient. Adipose tissue can also secrete pro-inflammatory cytokines that directly regulate cardiac electrical properties [16]. An additional factor that modifies the QT interval is autonomic tone. For instance, autonomic blockade prolongs the QT interval in normal subjects [17], and left stellate ganglion stimulation can modify the regional dispersion of ventricular refractoriness, promote triggered activity, and reduce the threshold for VF [18].

In contrast to the prominent role of EADs in the initiation of TdP in LQTS, polymorphic VT and VF in CPVT are triggered by delayed afterdepolarizations (DADs) that occur during the diastolic phase of the cardiac cycle due to intracellular calcium overload. DADs are typically caused by the spontaneous release of calcium from the sarcoplasmic reticulum, leading to a depolarizing inward sodium–calcium exchange current (I_NCX_) [19].

In BrS, VF is a result of complex interactions between genetic mutations in the SCN5A gene, leading to reduced sodium channel function and unique electrophysiological properties of the right ventricular epicardium, which has a large I_to_. As I_to_ is prominent in the subepicardium but not in the subendocardium, a reduced sodium current may allow a large unopposed I_to_ to prematurely repolarize the cell membrane potential and lead to very short APDs in the subepicardium compared to the subendocardium [20]. This non-physiological transmural voltage gradient can create phase 2 reentry and triggered activity, which contributes to the arrhythmogenic substrate in BrS [21]. In contrast to other channelopathies, which are typically not associated with structural heart disease, fibrosis in the RVOT has been implicated as the arrhythmic substrate in BrS [22].

As the path to VF in channelopathies is shaped by the specific channelopathy and environmental factors, it is unlikely that a single ablation technique is curative. However, by altering the underlying electrical abnormalities that predispose patients with channelopathies to VF, ablation may reduce the frequency and severity of arrhythmic events and improve outcomes in selected patients. The following sections will explore the rationale behind ablation strategies in the context of BrS, LQTS, CPVT, and SQTS highlighting the challenges and opportunities associated with this evolving field of cardiac electrophysiology.

## 3. Brugada Syndrome

BrS is an autosomal dominant channelopathy with variable expression that was first defined in 1992 in a group of eight patients with a common ECG phenotype, recurrent VF, and aborted SCD [23]. The type I Brugada pattern is associated with coved ST-segment elevation ≥2 mm followed by an inverted T-wave in the right precordial leads, while the type II and III Brugada patterns have ST-segment elevations with a saddleback appearance followed by a positive or biphasic T-wave. Only the type I pattern is diagnostic for BrS, while types 2 and 3 are considered suggestive of the disease. The prevalence of the Brugada pattern on ECG varies from 0.1% to 1% depending on the geographical region [24,25,26,27]. The global pooled prevalence of BrS has been estimated to be 0.5 per 1000 [28]. This is likely to be an underestimate of the actual prevalence due to the presence of asymptomatic patients, dynamic ECG changes, and the fact that many victims of SCD remain undiagnosed. People of Asian descent are disproportionately affected, with the highest prevalence of BrS found in Southeast Asia [28,29]. Furthermore, males may have an up to eight times higher prevalence of the Brugada ECG pattern and event rate than females [30,31,32,33]. The exact mechanism for the variation remains unclear, however, modulation of the ion channels by testosterone in the epicardium has been suggested as a probable mechanism [34]. The age of diagnosis is usually adulthood with the mean age around 41 years [35]. The first genetic alteration was recognized in 1998 in *SCN5A*, which encodes for the cardiac sodium channel [36]. To date, it remains the primary gene associated with BrS, with more than 350 loss-of-function variants, which are identified in approximately 30% of cases [37]. Many other genetic variants in genes such as *SCN10A*, *HEY2*, and *GPD1-L*, among others, have also been reported in BrS; however, their link with causality is unclear [38,39,40,41]. Autonomic system involvement in the form of imbalance of the parasympathetic and sympathetic nervous system has been reported as an important part of arrhythmogenesis in BrS, explaining the increased incidence of VF and SCD during nocturnal hours and with parasympathomimetic drugs in these patients [42,43].

The diagnosis is based on ECG changes, medical history, clinical symptoms, family history, and genetic tests [34,44]. Although BrS has primarily been associated with a structurally normal heart, there is growing evidence of minor structural changes including fibrosis in the RVOT [45]. Interestingly, two independent studies identified a small subset of patients with BrS who present with monomorphic VT, with RVOT origin being the most common, followed by bundle branch reentrant VT [46,47]. It is likely that the fibrosis in the RVOT seen on cardiac MRI in patients with BrS [45] favors the development of RVOT VT while the infrahisian conduction disease associated with *SCN5A* variants may predispose to bundle branch reentrant VT. In these studies, endocardial ablation of monomorphic VT was highly effective.

Prevention of VF in BrS focuses on avoiding precipitating medications (www.brugadadrugs.org; accessed on 15 January 2024.) and prompt treatment of fever. Currently, ICD implantation is recommended in patients at the highest risk for SCD, such as those with a history of aborted SCD or syncope secondary to VA [4]. The reduced function of the sodium channel leads to the loss of the action potential dome predominantly in the epicardial cells, which possess a large transient outward current, causing phase 2 re-entry [48]. The largest I_to_ densities are found in the epicardial RVOT, which may explain the right precordial ST-elevations characteristic of BrS.

Experimental studies suggest that epicardial ablation by radiofrequency ablation in the RVOT is more effective than targeting the endocardium. In an experimental model of BrS, Morita et al. mapped electrical activity on the epicardial and or transmural surface in 17 arterially perfused canine right ventricle preparations. This study concluded that all successful VT or PVC-eliminating radiofrequency ablations (RFA) were confined to the epicardial region and none of the endocardial ablations were successful [49]. Early clinical studies in BrS patient cohorts confirmed that CA may be useful in reducing the number of ICD shocks in highly selected BrS patients with a history of recurrent VF episodes. Nademanee et al. first reported the findings of nine BrS patients with a history of VF who underwent an EP study and subsequent CA. Electroanatomic mapping (EAM) demonstrated abnormally low, prolonged voltages and fractionated late potentials localized to the anterior part of the RVOT epicardium. CA of these sites resulted in non-inducibility of VT/VF in seven of the nine patients (78%) and normalization of the Brugada ECG pattern in eight patients (89%). The outcomes were favorable with no recurrence of VT/VF in eight of the nine patients (89%) after a follow-up period of 20 ± 6 months and only one patient was resumed on amiodarone after VF recurrence and had no further episodes at 33 months [50]. Pappone et al. reported that RFA of the substrate localized in the RV epicardium rendered VF/VT non-inducible in 135 patients with symptomatic BrS. This treatment also resulted in the normalization of their ECG [51]. Similar findings were reported in the BRAVO trial (Brugada Ablation of VF Substrate Ongoing Multicenter Registry), which included 159 symptomatic BrS patients with a history of cardiac arrest or syncope with documented episodes of spontaneous VF. Patients with overt structural heart disease were excluded from the study. A total of 140 [88%] patients experienced numerous ICD shocks for VF. A total of 151 of the 159 patients underwent EAM with percutaneous epicardial substrate ablation and the remaining 8 underwent ablation via open thoracotomy. The epicardial substrate in the RVOT was reported in all, and 81% of the patients had no recurrence of VT/VF after a single ablation, with the success rate rising to 96% after a subsequent procedure during a mean follow-up duration of 48 ± 29 months from the last ablation [52]. There was a significant reduction in the VF burden and frequency of shocks from 1.1 ± 2.1 per month before ablation to 0.003 ± 0.14 per month after the last ablation (*p* < 0.0001). Notably, normalization of the type 1 Brugada ECG pattern with or without sodium channel blockade after ablation was associated with favorable VF-free outcomes [52]. There is a paucity of data on the endocardial ablation approach in BrS patients. Talib et al. reported findings of 21 patients who underwent endocardial ablation. In 81% of patients, endocardial mapping did not identify an arrhythmogenic substrate in the endocardium. Among the patients who underwent ablation, one-third of the patients experienced VF recurrence during the mean 55-month follow-up period [53]. Similar findings were reported in a systematic review, which found that in 92.9% of the patients who underwent combined epicardial and endocardial mapping, no arrhythmogenic substrate was identified in the endocardium and 89.3% had substrates exclusively in the epicardium [54]. The rates of VF recurrence were higher at 29.4% in patients who underwent endocardial-only mapping with substrate modification compared to 3.3% among patients who underwent epicardial mapping with substrate modification [54].

We believe that ablative strategies should be reserved for high-risk BrS patients such as those experiencing VF cardiac arrest, VF storm, or recurrent ICD shocks despite optimal medical therapy. Nonetheless, international guidelines indicate that medical therapy should be attempted prior to ablation therapy, and the use of quinidine or hydroquinidine precedes the use of ablation therapy in the management of patients with BrS [55]. Lastly, CA is not recommended in asymptomatic BrS patients, irrespective of the predicted risk. Studies using ablation in patients with BrS are summarized in Table 1.

## 4. Congenital Long QT Syndrome

Congenital LQTS is an inherited arrhythmogenic syndrome characterized by a prolonged QT interval secondary to ion channel dysfunction, which increases the risk of life-threatening arrhythmias [64]. The prevalence of congenital LQTS is estimated to be 1:2000 [65]. It is caused by mutations in myocardial ion channel genes and has an autosomal dominant inheritance. The most prevalent genotypes are LQT1, LQT2, and LQT3, corresponding to mutations in the *KCNQ1*, *KCNH2*, and *SCN5A* genes, respectively. QT prolongation in LQTS is due to a prolonged cardiac APD caused by decreased repolarizing I_Ks_ or I_Kr_ activity or persistent sodium influx extending through the plateau phase. Ion channel dysfunction predisposes ventricular myocytes to EADs responsible for TdP and life-threatening VF [5]. The Purkinje network and myocardial fibers of the RVOT have been proposed as the primary sites for EADs triggering TdP [13].

The management of LQTS primarily includes lifestyle changes, avoidance of QT-prolonging drugs (www.crediblemeds.org; accessed on 15 January 2024), and administration of nonselective β-blockers. Sympathectomy and ICD implantation are generally reserved for refractory cases or patients intolerant to β-blockers. The efficacy of medical treatment differs based on the type of LQTs. Patients with LQTS1 respond best to β-blockers, and non-selective β-blockers such as nadolol or propranolol are deemed superior to β_1_-selective β-blockers [66]. Sodium channel blockers such as mexiletine are indicated for LQTS3 [67]. RFA has been proposed as a treatment method for LQTS, with promising results in small-scale studies. Haïssaguerre et al. reported the successful elimination of triggers arising from the Purkinje system and RVOT by RFA in four patients with LQTS. Over a 17-month follow-up, none of the patients had a recurrence of symptomatic VAs, though one patient had persistent premature ventricular contractions (PVCs) [13]. More recently, Pappone et al. reported successful ablation of VA substrates in the right epicardial region with no recurrence after a 12-month follow-up in 11 symptomatic LQTS patients who experienced recurrent ICD shocks before RFA. The mean age of the subjects was 44.0 ± 7.8 years [14]. However, the lack of larger studies to support the generalizability of the results and the lack of evidence of structural abnormalities in patients with LQTS limits the use of RFA in these patients [68,69]. Consequently, CA has an experimental nature in LQTS patients in select centers and is not part of the management pathway for LQTS. Further long-term studies are necessary to provide a greater understanding of the effectiveness and safety of the procedure. Studies using ablation in patients with LQTS are summarized in Table 2.

## 5. Catecholaminergic Polymorphic Ventricular Tachycardia

CPVT is a rare familial arrhythmogenic disorder that generally presents during childhood or adolescence and is characterized by polymorphic VT, triggered by physical or emotional stress in a structurally normal heart. The prevalence of the disease is estimated to be 1:10,000 [70]. Males present with symptoms earlier and are more predisposed to cardiac events [71]. Various genes associated with CPVT include the cardiac ryanodine receptor gene (*RYR2*), and the calmodulin genes (*CALM1, CALM2, CALM3*), which have autosomal dominant inheritance. Mutations in calsequestrin 2 (*CASQ2*), triadin (*TRDN*), and trans-2,3-enoyl-CoA reductase-like (*TECRL*) follow an autosomal recessive pattern of inheritance [72]. The genes involved in CPVT are generally associated with intracellular calcium homeostasis, and their genetic variants lead to DAD-mediated triggered activity, causing frequent PVCs, polymorphic VT, and VF [73]. Supraventricular arrhythmias, such as atrial ectopic beats, atrial fibrillation, and nonsustained supraventricular tachycardia, have been reported in patients with CPVT, and these can act as a trigger for VT/VF [74].

Antiadrenergic drugs, such as β-blockers combined with flecainide, remain the mainstay therapy in the management of CPVT. Refractory cases benefit from sympathectomy to minimize the risk of VA. A systematic review analysis of 503 patients reported inappropriate shocks and electrical storms in approximately 20% of patients, and death due to an ICD-related electrical storm in four patients [75]. Notably, the bidirectional VT in patients with CPVT is less responsive to ICD shocks, emphasizing the need for careful patient selection before considering ICD implantation [76]. Thus, implantation of ICDs should be reserved for the most severe cases, due to the proarrhythmic risk of inappropriate shocks, triggering an electrical storm. Additionally, device-associated complications have been reported in approximately one-third of the patients with channelopathies [75]. Importantly, the young age at presentation heightens the risk of device-related complications including ICD lead failure and frequent generator changes increasing the risk of infections. Furthermore, younger individuals tend to have more active lifestyles, which increases the mechanical stresses placed on the ICD system. Physical activities and vigorous movements can lead to lead fractures, lead dislodgements, or damage to the device or leads.

CA has not been well-studied in CPVT and medications, including non-selective β-blockers and flecainide, as well as sympathectomy for refractory cases remain the cornerstone of CPVT management. The polymorphic nature of PVCs with adrenergic stress makes PVCs a less attractive target for ablation. Shen et al. reported outcomes of CA in 14 Chinese CPVT patients with recurrent syncope on maximally tolerated β-blockers who were unable to receive flecainide [77] Of note, these patients were on propranolol or metoprolol, as nadolol is unavailable in China. These patients underwent CA of the VA-triggering PVCs. A total of 26 triggering ectopic beats were recorded: 9 exhibited right bundle branch block configuration with inferior axis, 8 revealed right bundle branch block configuration with superior axis, 4 showed left bundle branch block configuration with inferior axis, and 5 exhibited left bundle branch block configuration with superior axis, confirming the polymorphic nature of PVCs arising from the right and left ventricles in CPVT. While 25/26 triggering PVCs were successfully ablated, after a 49-month follow-up period, only 50% remained free of recurrent syncope, which was attributed to the persistent presence of non-triggering PVCs post-ablation [77]. Kaneshiro et al. reported long-term outcomes in a case series of five patients who underwent RFA of premature ventricular beats triggering VF. Four of the five patients did not achieve complete suppression of VA during long-term follow-up (mean duration, 71 ± 29 months). However, there was a decrease in the number of VA events, and the average duration from ablation to recurrence of VA was approximately 4 years. Additionally, one patient experienced an electrical storm resulting from an inappropriate shock for atrial fibrillation, which was effectively managed by pulmonary vein isolation [78]. Kawada et al. reported outcomes of CA in a CPVT patient with refractory atrial tachyarrhythmias. After completion of pulmonary vein isolation, isoproterenol infusion induced polymorphic VT as well as multifocal AT originating mostly from the right atrium. After ablation of the atrial tachycardia site patient was discharged on bisoprolol and flecainide. After one year of follow-up, no syncopal events, exercise-induced VA, or atrial tachycardias were observed [79]. These studies highlight the importance of ablation of atrial tachyarrhythmia in patients with CPVT to minimize the risk of triggers for VA.

## 6. Short QT Syndrome

SQTS is a rare, autosomal dominant channelopathy characterized by a short QT interval associated with SCD. A gain-of-function mutation in potassium outward currents and a loss-of-function mutation in genes encoding calcium inward currents results in a shortened APD predisposing to reentrant atrial and VAs [3]. Patients with a history of VF require an ICD to prevent SCD and in patients with recurrent shocks from VF, quinidine has proven effective in reducing the risk of recurrent VF. There is a paucity of data on outcomes in patients with SQTS undergoing CA. Morimoto et al. reported a case of a 50-year-old woman who underwent ICD implantation after an aborted SCD and had persistent PVCs and short QT refractory to quinidine and sotalol therapy. She underwent a successful RFA targeting a PVC from the inferolateral RV, which triggered VF. During a 17-month follow-up period, no arrhythmic events were reported [80]. These findings underscore CA being a possible therapeutic strategy in patients with SQTS with persistent VA, and recurrent ICD shocks despite antiarrhythmic therapy.

## 7. Conclusions

With the rapid advancements in arrhythmia mapping and CA technologies, the role of ablation therapy is expanding to diseases where its potential has not been explored. Several studies have demonstrated the effectiveness of epicardial CA in the RVOT in reducing recurrent VF events in BrS patients with a history of VF, which suggests that CA should be integrated into a multimodality treatment paradigm for these high-risk BrS patients. In LQTS, CPVT, and SQTS, CA is less studied, and a significant therapeutic potential has not been established. We therefore advocate for careful and informed decisions regarding the selection of candidates for CA and an evidence-based approach to defining optimal clinical management pathways for patients with cardiac channelopathies.

## Figures and Tables

**Table 1:** Studies using ablation in patients with Brugada syndrome.

No.	First Author, Year	Study Design	Total Population		Repeat Ablation	Notable Points
			Ablated	Not Ablated		
1.	Haïssaguerre, 2003 [13]	Cohort Retrospective	3	0	0	PVC triggers successfully ablated from endocardial RVOT (n = 2) RV and Purkinje fibers (n = 1).
2.	Sunsaneewiyatakul, 2012 [56]	Cohort Retrospective	4	6	0	Endocardial RVOT ablation. No more VF storm at 12–30-month follow-up.
3.	Brugada, 2015 [57]	Cohort Prospective	14	0	0	RV epicardial ablation (anterior RV free wall and RVOT) of low voltage areas identified with mapping before and after flecainide. VA not inducible after 5-month follow-up.
4.	Rodríguez-Mañero, 2015 [46]	Cohort Retrospective	6	828	0	4.2% of the patients of BrS with ICD experienced monomorphic VT: Successful ablation of RVOT tachycardia (from the endocardium, n = 4 and epicardial and endocardial ablation, n = 1), endocardial lateral mitral annulus, n = 1, and BBRVT, n = 2.
5.	Zhang, 2016 [58]	Cohort Prospective	11	0	0	Despite epicardial RVOT ablation, 27% of the patients experienced recurrence of VA.
6.	Chung, 2017 [59]	Cohort Retrospective	15	0	1	Flecainide provocation test, n = 15, and epicardial warm water instillation (n = 6) led to enhancement of functional epicardial substrates. Successful ablation in 14 of 15 patients; 1 patient required repeat ablation with no further recurrence of VA at 1-year follow-up.
7.	Pappone, 2017 [51]	Cohort Prospective	135	0	2	Ajmaline provocation test. Arrhythmogenic electrophysiological substrate commonly present in the RV epicardium.
8.	Shelke, 2017 [60]	Cohort Retrospective	5	0	0	Substrate modification of RVOT was performed endocardially in one patient, both endocardial and epicardial in three, and only epicardially in one patient. One patient had an episode of VF at 24 months after ablation.
9.	Talib, 2023 [53]	Cohort Retrospective	21	102	0	Endocardial ablation of PVCs triggering VF storm from RVOT (n = 18) and RV anterior free wall (n = 2), RV inflow tract (n = 1). Notching of QRS in lead V_1_ was associated with high VF recurrence.
10.	Tokioka, 2019 [47]	Cohort Retrospective	7	181	0	Ablation in BrS patients with monomorphic VT: RVOT (n = 4), tricuspid annulus (n = 1), LV septum (n = 1), BBRVT (n = 1). One patient had recurrent VT.
11.	Kamakura, 2021 [61]	Cohort Retrospective	16	0	0	Ajmaline provocation. Abnormal epicardial potentials in RVOT in all 16 patients and in the epicardial RV inferior wall in 12/16 patients. Combined endocardial and epicardial ablation was suggested, with endocardial ablation as an alternative to epicardial ablation for ablating areas close to the coronary artery. No patients had recurrent VF storm after ablation.
12.	Mamiya, 2021 [62]	Cohort Retrospective	11	16	0	Pilsicainide provocation test. Epicardial RVOT ablation. Improvements in the ECG parameters were associated with decreased VF recurrence post-procedure. A total of 4/11 patients had recurrent VF after ablation.
13.	Haïssaguerre, 2022 [63]	Cohort Retrospective	17	2	3	Most common triggers were located in the epicardial RVOT and epicardial inferior RV; epicardial ablation in all patients, and combined endo-epi ablation in 2/17. A total of 6/17 patients had recurrent VF.

BBRVT = bundle branch reentry ventricular tachycardia, BrS = Brugada syndrome, ECG = electrocardiogram, ICD = implantable cardioverter defibrillator, PVC = premature ventricular contraction, RV = right ventricle, RVOT = right ventricular outflow tract, VA = ventricular arrhythmias, VT = ventricular tachycardia, VF = ventricular fibrillation.

**Table 2:** Studies using ablation in patients with long QT syndrome.

No.	First Author, Year	Study Design	Total Population		Repeat Ablation	Notable Points
			Ablated	Not Ablated		
1.	Haïssaguerre, 2003 [13]	Cohort Retrospective	4	0	0	PVC triggers successfully ablated from LV Purkinje fibers (n = 3) and endocardial RVOT (n = 1).
2.	Pappone, 2023 [14]	Cohort Prospective	11	0	0	Endo- and epicardial mapping of RV and LV. Ablation of structural electrophysiological abnormalities in the epicardium of RV prevents recurrences.

RV = right ventricle, RVOT = right ventricular outflow tract, VF = ventricular fibrillation.

## Data Availability

Data are contained within the article.
